# Breast cancer management pathways during the COVID-19 pandemic: outcomes from the UK ‘Alert Level 4’ phase of the B-MaP-C study

**DOI:** 10.1038/s41416-020-01234-4

**Published:** 2021-03-25

**Authors:** Rajiv V. Dave, Baek Kim, Alona Courtney, Rachel O’Connell, Tim Rattay, Vicky P. Taxiarchi, Jamie J. Kirkham, Elizabeth M. Camacho, Patricia Fairbrother, Nisha Sharma, Christopher W. J. Cartlidge, Kieran Horgan, Stuart A. McIntosh, Daniel R. Leff, Raghavan Vidya, Shelley Potter, Chris Holcombe, Ellen Copson, Charlotte E. Coles, Ramsey I. Cutress, Ashu Gandhi, Cliona C. Kirwan, on behalf of the B-MaP-C study collaborative, Amit Agrawal, John Benson, Parto Forouhi, Primeera Wignarajah, Anu Shrotri, Arjun Kattakayam, Jarin Louis Noronha, Lee Martin, Mohamed Lafi, Rob Hardy, Khalid Amin, Abdalla Saad Abdalla AL-Zawi, Mohamed Elamass, Ali Salih, Firas Eddin Bachir Alkistawi, Anna Heeney, Arnold D. K. Hill, Colm Power, Michael J. Allen, Ashok Chouhan, Rathi Rathinaezhil, Samy Shaheed, Charles Zammit, Gillian Clayton, Sascha Dua, Simon Smith, Tasha Gandamihardja, Chloe Williams, Donna Egbeare, Eleri Davies, Helen M. Sweetland, Sharat Chopra, Sumit Goyal, Dalia Elfadl, Dheer Singh Rana, Eliana Kalakouti, Musa Barkeji, Rajiv Vashisht, Ralia Bunza, Saung Hnin Phyu, Ciaran Hollywood, Iman Azmy, Julia Massey, Anita Hargreaves, Claudia Harding-Mackean, Jane Ooi, Joanna Seward, Helen Mathers, Norah Scally, Reem Salman, Hyunjin Shin, Jane Turner, Lubna Noor, Sanjay Joshi, Sarah Horne, Wail Al Sarakbi, Peter Liptay-Wagner, Rosamond Jacklin, Sankaran Chandrasekharan, Simon Marsh, Sunita Saha, Christopher Wilson, Claire Louise Rutherford, Julie Doughty, Laszlo Romics, Sheila Stallard, Anushka Chaudhry, Jennifer Peck, Nathan Coombs, Samantha K. Williams, Simon Hawkins, Ashutosh Kothari, Hisham Hamed, Urvashi Jain, Ian Daltrey, Nick Abbott, Russell Mullen, Dorin Dumitru, Eiman Khalifa, Masuma Sarker, M. Bilal Elahi, Raouef Ahmed Bichoo, Anzors Gvaramadze, Dinesh Thekkinkattil, Jibril Jibril, Demetrios Hadjiminas, Edward R. C. St John, Henry Douglas Robb, Katy Hogben, Nur Amalina Che Bakri, Paul Thiruchelvam, Georgios Exarchos, Ragheed Al-Mufti, Caroline Mortimer, Evangelos Mallidis, Georgios Karagiannidis, Hussein Tuffaha, Inga Peerlink, Rajive Nair, Dinesh Thekkinkattil, Lydia Prusty, Anzors Gvaramadze, Jibril Jibril, Amtul Sami, Alex Knight, Duraisamy Ravichandran, Katharine Kirkpatrick, Ruth James, Deepika Akolekar, Disha Mehta, Ellora Barua, Hannah Knowles, Haresh Devalia, Karina Cox, Mohsin Dani, Ritchie Chalmers, Anjana Satpathy, Edel Quinn, Gerard Byrne, James Harvey, John Murphy, Lyndsey Highton, Mohammad Amir Sharif, Nicola Barnes, Nikitas Dimopoulos, Richard Johnson, Sumohan Chatterjee, Hiba Fatayer, Vinod Mathen, Amanda Taylor, Rachel Soulsby, Adam Walsh, Amanda Thorne, Jasper Gill, Louise Merker, Adam Critchley, Andrew Pieri, Henry Cain, Jane Ralph, Loraine Kalra, Robert Thomas, Ian Young, Lucy R. Khan, Beatrix Elsberger, Elizabeth Smyth, Gordon Urquhart, Mairi Fuller, Yazan Masannat, Ada Chrysafi, Muhammad Salman, El-Rasheed Abdalla, Katalin Zechmeister, Maged Hussien, Mina M. G. Youssef, Angeline Tanhueco, Reginald Salvador, Sharon Wallace, Simon Pain, Ajay Sahu, Alice Chambers, Alice Moody, Isabella Dash, James Cook, Jeremy Batt, Michelle Mullan, Mike Shere, Nicholas Gallegos, Rachel Ainsworth, Sasi Govindarajulu, Shelley Potter, Zenon Rayter, Kate E. Williams, Maria Bramley, Mohammed Absar, Nabila Nasir, Rami Tabbakh, Bernadette Pereira, Jasdeep Gahir, Karen Bosch, Oladapo Fafemi, Nader Touqan, Georgette Oni, Hazem Khout, Kristjan Asgeirsson, Lisa Whisker, Rachel Xue Ning Lee, Robert Macmillan, Stephen McCulley, Tuabin Rasheed, Asha Adwani, Ashvina Segaran, David Dodwell, Dennis Remoundos, Gael MacLean, Giulio Cuffolo, Michael Douek, Pankaj Roy, Toral Gathani, Mohammed Absar, Erum Najeeb, Claudiu Simonca, Maria Verroiotou, Sa’ed Ramzi, Stephanie C. Jenkins, Vallipuran Gopalan, Sarah Barker, Ciara McGoldrick, Gareth W. Irwin, Peter Mallon, Samantha A. Sloan, Abbas Imran, Giuseppina Mondani, Iain Brown, Imran Abbas, Mona Sulieman, Philip Drew, Polly King, Rachel Elizabeth English, Anita Sharma, Charlotte Ives, Douglas Ferguson, George Boundouki, James Bentley, Jenny Banks, Julie Dunn, Rachel Tillett, Sisse Olsen, Anne Tansley, Emma de Sousa, Geraldine Mitchell, Ian Whitehead, Julia Henderson, Matthew Rowland, Mysore Chandrashekar, Raja Eid, Elizabeth Clayton, Farrokh Pakzad, Jonathan D. Horsnell, Matthew Hague, Polly Partlett, Tracey Irvine, Charlotte Kallaway, Katherine Fairhurst, Christiana Laban, Jamie McIntosh, Nicola Laurence, Richard Sutton, Anup Sharma, Dibyesh Banerjee, Nadine Betambeau, Sabrina Bezzaa, Sonia Bathla, Atanu Ray, Leena Chagla, Tamara Kiernan, Brian Hogan, Channegowda Navin, Emma Macinnes, Philip Turton, Raj Achuthan, Venla Kantola, Shireen Mckenzie, Helen Dent, Caroline Pogson, Shamaela Waheed, Tania S. de Silva, Usama Suleiman, Lucie Jones, Ruvinder Athwal, Simon Harries, Catherine Krzyzanowska, Abeera Abbas, Anna R. Hurley, Gerald Gui, Jennifer E. Rusby, Katherine Krupa, Kathryn E. Harborough, Nicola Roche, Peter A. Barry, Rebekah Law, William H. Allum, Cheryl Lobo, Eleni Ntakomyti, Joanna Franks, Massimiliano Cariati, Neill Patani, Noyko Stanilov, Petros Charalampoudis, Zarghuna Taraki, Kat McEvoy, Mohamed Razick Sait, Stuart Robertson, Bashar Zeidan, David Rew, Fayyaz Mazari, Louise Alder, Vasileios Sakellariou, Ahmed Hamad, Amit Goyal, Amtul Carmichael, Carol-Ann Courtney, David Mark Sibbering, Emanuele Garreffa, Kwok-Leung Cheung, Susan Williams-Jones, Yasmin Wahedna, Aonghus Ansari, Frances Kenny, Kalliope Valassiadou, Kelly Lambert, Jaroslaw Krupa, Mini V. Sardar, Monika Kaushik, Sheila Shokuhi, Simon Pilgrm, Walid Sasi, Penelope McManus, Rishikesh Parmeshwar, Santosh Somasundaram, Manoj Gowda, Sadaf Jafferbhoy, Sankaran Narayanan, Sekhar Marla, Soni Soumian, Ngee-Ming Goh, Jamie Vatish, Tin Aung Sein, Ennio Agabiti, Joseph Maalo, Kelvin Chong, Lee-Min Lai, Mohamed Elkorety, Sherif Monib, Simon Thomson, Youhana Mikhael, Bahar Mirshekar-Syahkal, Jane Aitken, Mina Girgis, Dibendu Betal, Fabio Rapisarda, Lorna Cook, Olubunmi Odofin, Riccardo Bonomi, Stacy Wardle, Wendy Sotheran, Irene Athanasiou, Jonathan Lund, Maria Callaghan, Rajaram Burrah, Raman Vinayagam, Karen James, Shabbir Poonawala, Brian Isgar, Pilar Matey, Senthurun Mylvaganam, Carl Podesta, Tapan Sircar, Fathi Salem, Zaid Al-Ishaq

**Affiliations:** 1 The Nightingale Breast Cancer Centre, Wythenshawe Hospital, Manchester University NHS Foundation Trust, Manchester, M23 9LT UK; 2 Department of Breast Surgery, St. James’s University Hospital, Leeds, LS9 7TF UK; 3 Department of Surgery and Cancer, Imperial College, London, UK; 4 Department of Breast Surgery, The Royal Marsden NHS Foundation Trust, Downs Road, Sutton, Surrey SM2 5PT UK; 5 Leicester Cancer Research Centre, Clinical Sciences Building, University of Leicester, Leicester, LE2 2LX UK; 6 Division of Population Health, Health Services Research, and Primary Care, School of Health Sciences, University of Manchester, Manchester, M13 9PL UK; 7 Trustee, Independent Cancer Patients Voice, Manchester, UK; 8 Breast unit, Level 1 Chancellor wing, St James’s Hospital, Leeds, LS9 7TF UK; 9 Queen Margaret Hospital, Dunfermline, Whitefield Rd, Dunfermline, KY12 0SU UK; 10 Patrick G Johnston Centre for Cancer Research, Queen’s University Belfast, 97 Lisburn Road, Belfast, BT9 7AE UK; 11 The Royal Wolverhampton NHS Trust, Wolverhampton Road, Wolverhampton, WV10 0QP UK; 12 Bristol Centre for Surgical Research, Population Health Sciences, Bristol Medical School, Canynge Hall, Whatley Road, Bristol, BS8 2PS UK; 13 Bristol Breast Care Centre, North Bristol NHS Trust, Southmead Road, Bristol, BS10 5NB UK; 14 Linda McCartney Centre, Royal Liverpool and Broadgreen University Hospital, Prescot Street, Liverpool, L7 8XP UK; 15 Cancer Sciences Academic Unit, University of Southampton and University Hospital Southampton, Tremona Road, Southampton, SO16 6YD UK; 16 Department of Oncology, University of Cambridge, Cambridge, UK; 17 Division of Cancer Sciences, School of Medical Sciences, Faculty of Biology, Medicine and Health, University of Manchester, Oglesby Cancer Research Building, Manchester Cancer Research Centre, Wilmslow Road, Manchester, M20 4BX UK; 18 Addenbrookes Hospital, Cambridge, United Kingdom; 19 Aintree University Hospital, Liverpool, United Kingdom; 20 Airedale NHS Foundation Trust, London, United Kingdom; 21 Basildon & Thurrock University Hospital, Basildon, United Kingdom; 22 Beaumont Hospital, Dublin 9, Dublin, Ireland; 23 Brighton and Sussex university hospitals NHS Trust, Brighton, United Kingdom; 24 Broomfield Breast Unit, Mid and South Essex NHS Foundation Trust, Broomfield, USA; 25 Cardiff and Vale University Health Board, Cardiff, United Kingdom; 26 Chelsea and Westminster NHS foundation trust, London, United Kingdom; 27 Chesterfield Royal Hospital, Chesterfield, United Kingdom; 28 Countess of Chester NHS Foundation Trust, Chester, United Kingdom; 29 Craigavon Area Hospital, Craigavon, United Kingdom; 30 Croydon University Hospital, Thornton Heath, United Kingdom; 31 East Suffolk and North Essex Foundation Trust, Colchester, United Kingdom; 32 East Suffolk and North Essex Foundation Trust (Colchester hospital), Colchester, United Kingdom; 33 Gartnavel General Hospital, Glasgow, United Kingdom; 34 Great Western Hospital NHS Foundation Trust, Swindon, United Kingdom; 35 Guy’s and St Thomas’ NHS foundation trust, London, United Kingdom; 36 Highland Breast Centre, Raigmore Hospital, Inverness, United Kingdom; 37 Hull University Teaching Hospitals NHS Trust, Hull, United Kingdom; 38 United Lincolnshire County Hospitals, Lincoln, United Kingdom; 39 Imperial College Healthcare NHS Trust, London, United Kingdom; 40 Ipswich Hospital NHS Trust, Ipswich, United Kingdom; 41 Lincoln County Hospital, Lincoln, United Kingdom; 42 Luton and Dunstable University Hospital, Luton, United Kingdom; 43 Maidstone & Tunbridge Wells NHS Trust, Tunbridge Wells, United Kingdom; 44 Manchester University NHS Foundation Trust, Manchester, United Kingdom; 45 Milton Keynes University Hospital, Milton Keynes, United Kingdom; 46 Musgrove Park Hospital, Taunton, United Kingdom; 47 Newcastle upon Tyne Hospitals NHS Foundation Trust, Newcastle upon Tyne, United Kingdom; 48 NHS Fife, Kirkcaldy, United Kingdom; 49 NHS Forth Valley Royal Hospital, Larbert, United Kingdom; 50 NHS Grampian, Aberdeen, United Kingdom; 51 Nobles Hospital Isle of Man, Isle of Man, United Kingdom; 52 Norfolk and Norwich University Hospital, Norwich, United Kingdom; 53 Norfolk and Norwich University Hospital, Norwich, United Kingdom; 54 North Bristol NHS Trust, Bristol, United Kingdom; 55 North Manchester Care Organisation, Manchester, United Kingdom; 56 North Middlesex University Hospital, London, United Kingdom; 57 Northern Care Alliance, Pulau Pinang, Malaysia; 58 Nottingham Breast Institute, Nottingham, United Kingdom; 59 Oxford University Hospital NHS Foundation Trust, Oxford, United Kingdom; 60 Pennine Acute Hospital NHS Trust, Pennine, United Kingdom; 61 Plymouth University Hopsitals NHS trust, Plymouth, United Kingdom; 62 Primrose Breast Care Centre, University Hospitals Plymouth NHS, Plymouth, United Kingdom; 63 Queen Elizabeth University Hospital, Glasgow, United Kingdom; 64 Queen’s University Belfast, Belfast, United Kingdom; 65 Royal Cornwall Hospital Truro, Truro, United Kingdom; 66 Royal Devon & Exeter Hospital, Exeter, United Kingdom; 67 Royal Liverpool University Hospital, Liverpool, United Kingdom; 68 Royal Surrey NHS Foundation Trust, Guildford, United Kingdom; 69 Royal United Hospital Bath, Bath, United Kingdom; 70 Royal United Hospital, Bath, United Kingdom; 71 St George’s Hospital, London, United Kingdom; 72 St. Helens and Knowsley Trust, Rainhill, United Kingdom; 73 St. James’s University Hospital, Leeds, United Kingdom; 74 Surrey and Sussex Healthcare, West Sussex, England; 75 South Warwickshire NHS Foundation Trust, Warwick, United Kingdom; 76 Taunton and Somerset NHS Foundation Trust, Taunton, United Kingdom; 77 The Royal Marsden NHS Foundation Trust, London, United Kingdom; 78 University College London Hospital, Bloomsbury, United Kingdom; 79 University Hospital Coventry and Warwickshire NHS Trust, Coventry, United Kingdom; 80 University Hospital Southampton, Southampton, United Kingdom; 81 University Hospitals of Derby and Burton NHS Foundation Trust, Derby, United Kingdom; 82 University Hospitals of Leicester, Leicester, United Kingdom; 83 University Hospitals of Morecambe Bay, Kendal, United Kingdom; 84 University Hospitals of North Midlands, Stoke-on-Trent, United Kingdom; 85 University Hospitals Plymouth NHS Trust, Plymouth, United Kingdom; 86 Warwick Hospital, Warwick, United Kingdom; 87 West Hertfordshire Hospitals NHS trust, Watford, United Kingdom; 88 West Suffolk NHS Hospital, Bury Saint Edmunds, United Kingdom; 89 Western Sussex Hospitals NHS Foundation Trust, Chichester, United Kingdom; 90 Whittington Health NHS Trust, Highgate, United Kingdom; 91 Wirral University Teaching Hospital, Birkenhead, United Kingdom; 92 Royal Wolverhampton NHS Trust, Wolverhampton, United Kingdom

**Keywords:** Breast cancer, Surgical oncology, Health care economics, Quality of life, Health policy

## Abstract

**Background:**

The B-MaP-C study aimed to determine alterations to breast cancer (BC) management during the peak transmission period of the UK COVID-19 pandemic and the potential impact of these treatment decisions.

**Methods:**

This was a national cohort study of patients with early BC undergoing multidisciplinary team (MDT)-guided treatment recommendations during the pandemic, designated ‘standard’ or ‘COVID-altered’, in the preoperative, operative and post-operative setting.

**Findings:**

Of 3776 patients (from 64 UK units) in the study, 2246 (59%) had ‘COVID-altered’ management. ‘Bridging’ endocrine therapy was used (*n* = 951) where theatre capacity was reduced. There was increasing access to COVID-19 low-risk theatres during the study period (59%). In line with national guidance, immediate breast reconstruction was avoided (*n* = 299). Where adjuvant chemotherapy was omitted (*n* = 81), the median benefit was only 3% (IQR 2–9%) using ‘NHS Predict’. There was the rapid adoption of new evidence-based hypofractionated radiotherapy (*n* = 781, from 46 units). Only 14 patients (1%) tested positive for SARS-CoV-2 during their treatment journey.

**Conclusions:**

The majority of ‘COVID-altered’ management decisions were largely in line with pre-COVID evidence-based guidelines, implying that breast cancer survival outcomes are unlikely to be negatively impacted by the pandemic. However, in this study, the potential impact of delays to BC presentation or diagnosis remains unknown.

## Background

### COVID-19 impact in the United Kingdom

The first case of the novel coronavirus SARS-CoV-2 (coronavirus disease 2019, COVID-19) was confirmed in the United Kingdom on January 30, 2020.^1^ As of August 4, 2020, COVID-19 has resulted in 17,918,582 confirmed cases and 686,703 deaths worldwide since its emergence in December 2019.^2^ Globally, the COVID-19 pandemic has significantly impacted healthcare delivery, including alterations in cancer care. On 16th March, the UK’s lockdown response was initiated in response to the United Kingdom reaching ‘Alert Level 4’ (transmission high or rising exponentially), with advice against ‘non-essential’ travel, social distancing and guidance on self-isolation.^3^ On 17th March, NHS England announced that all non-urgent operations in England would be postponed from 15th April to free up 30,000 beds.^4^ The ‘Alert Level 4’ subsided on 8th May, with the relaxation of the ‘stay at home’ message.

### Management of breast cancer in the United Kingdom

There are 55,200 new breast cancer diagnoses per year, which represents 15% of all cancers diagnosed in the United Kingdom.^5^ Multi-modality treatment, including surgery, radiotherapy (RT) and systemic therapy, involves multiple hospital visits increasing the potential risk of exposure to COVID-19. With current treatments, early breast cancer prognosis is usually excellent.^5,6^ Compromises to cancer care during the COVID-19 pandemic as a result of rationalisation of resources and prioritisation of individual patient’s cancer versus COVID-19 risks have the potential to impact on survival, as well as the quality of life (QoL), service provision and health economics.

### Guidelines for the management of breast cancer during the COVID-19 pandemic

Multidisciplinary UK guidelines, as well as several European and American guidelines, were published early in the alert phase, informing management of breast cancer during the pandemic (Supplementary Tables 1 and 2).^7–13^ All aimed to assist rationalisation and prioritisation of delivery of breast services whilst healthcare resources were limited and hospitals were considered a high-infection risk environment. The majority of recommendations did not deviate substantially from pre-COVID National Institute for Health and Care Excellence (NICE) national guidance.^6^ Guidance emphasised multidisciplinary management, balancing the risk of COVID-19 infection during treatment and the burden of the COVID-19 pandemic on re-structured health services^7^ to enable the delivery of cancer care whilst safeguarding resources for patients with COVID-19 infection.

When theatre capacity was compromised, guidelines included the use of preoperative, or ‘bridging’, endocrine therapy (ET)^14^ and priority-driven management based on tumour biology^10,13,15^ (Supplementary Table 1) with the postponement of non-urgent surgery. Advice included reserving neoadjuvant chemotherapy (NACT)^13^ for non-operable disease only, careful consideration of the risk/benefit of chemotherapy and streamlined use of adjuvant radiotherapy (RT),^16^ including potential omission of breast or nodal radiotherapy or use of five-fraction radiotherapy (5F RT). The FAST-Forward trial results, published on April 28, 2020, demonstrated non-inferiority for local recurrence for 5F RT compared to the UK standard of care of 15 fractions (15F) and with improved early and similar late normal tissue toxicity,^17^ providing evidence for oncologically appropriate RT delivery in a reduced number of visits. There was a move towards creating ‘green’ operating capacity, defined as an operating theatre intended to be COVID-19 free (e.g., theatres geographically separate from patients treated for COVID-19, only doing elective cases with preperative negative SARS-CoV-2 test as a requirement). This is in contrast to a ‘red’ site, defined as an operating theatre delivering emergency surgery in hospitals caring for COVID-19 patients or patients without a preoperative negative COVID test. In the United Kingdom, this involved either restructuring of hospital facilities or sourcing operative capacity in the independent sector.

With the aim of minimising surgical complexity, length of stay and complication risks, and therefore reducing the risk of COVID-19 infection, immediate breast reconstruction (IBR) was suspended, with delayed reconstruction to be offered once services returned to normal. In addition, breast units across the United Kingdom suspended breast screening from March 2020.

### Aims and objectives

The aim of the B-MaP-C study was to determine (i) changes to breast cancer management during ‘UK alert level 4’ of the UK COVID-19 pandemic (March 16, 2020 to May 8, 2020),^6^ (ii) the potential repercussions of these changes to care in terms of oncological impact, quality of life (QoL) and healthcare costs and (iii) the impact of a concurrent COVID-19 diagnosis on patients undergoing treatment for breast cancer.

## Methods

A multicentre national cohort study was conducted in which consecutive patients with a diagnosis or early breast cancer undergoing MDT-guided treatment recommendations during the peak of the COVID-19 pandemic were eligible for inclusion.^18^ Full study information is available on bmapc.org. Patients were identified prospectively by the local participating clinical teams during the UK’s ‘Alert Level 4’ phase of the COVID-19 pandemic (defined as 16th March (the commencement of social distancing recommendations in the United Kingdom) to 8th May [relaxation of the ‘stay at home’ message]).

Data were collected and managed using REDCap™ electronic data capture tools hosted at The University of Manchester,^19^ in accordance with Caldicott II principles. Each participating unit was required to register the study locally with their hospital audit department and obtain local governance approvals prior to the commencement of data collection. Ethics approval was not required according to the NHS Health Research Authority online decision tool (www.hra-decisiontools.org.uk/research/).^18^

**Fig. 1:**
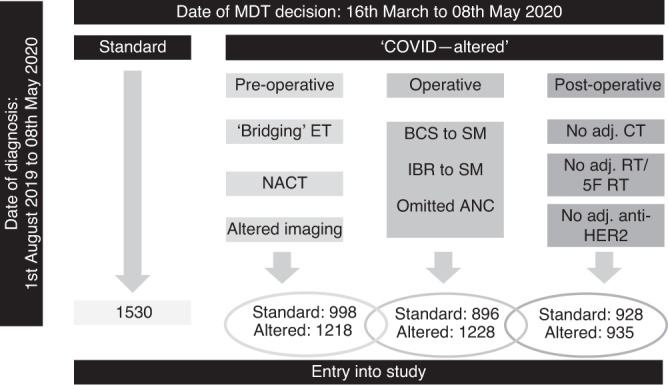
Study schematic showing the points at which patients may enter into the study, the number of patients in each group and the timelines reported in the study. Patients may have ‘standard’ treatment at all stages in their management journey or may have ‘COVID-altered’ treatment. This latter group may have ‘COVID-altered’ management in the preoperative, operative or post-operative stage, and may indeed have ‘standard’ management decision at some stage. Overlapping ovals indicate overlapping sub-cohorts. ET   endocrine therapy, NACT   neoadjuvant chemotherapy, BCS   breast-conserving surgery, SM   simple mastectomy, Adj. adjuvant, CT   chemotherapy, RT   radiotherapy, HER2   human epidermal growth factor receptor.

We collected patient demographic data, cancer-specific data and multidisciplinary treatment recommendations in the preoperative, operative and post-operative setting. Patients on NACT prior to the pandemic could enter the study period at the peri- or post-operative multidisciplinary team meeting (MDT-M) (Fig. 1), hence the inclusion of patients having a diagnosis from August 1, 2019. For each management decision, collaborating units determined whether the decision was ‘standard' (i.e., the same as would have been made pre-COVID) or ‘COVID-altered', i.e., not standard management for that unit’s usual practice (even if standard practice for other units). An example would be if a unit’s standard practice is to perform axillary node clearance after positive sentinel node, but during the alert level 4 period, a patient was recommended no further axillary surgery after positive sentinel node.^18^ Hence, any alterations in management identified were as a direct result of the COVID-19 pandemic, representing treatment out of the ordinary for that collaborating unit. This allowed us to reflect the changes caused by the pandemic, whilst taking into account the background variability of practice across the United Kingdom. Altered management decisions were interrogated in more detail and compared to pre-COVID NICE guidance on breast cancer management^6^ as well as published COVID-specific guidelines.^9,12,13,16,20^

### Data analysis

The study was reported in accordance with the STROBE guidelines for observational studies.^21^ A pre-specified statistical analysis plan was approved by the study steering group. The descriptive analysis examined characteristics of those patients in whom standard management was followed and those with ‘COVID-altered’ management. The subsequent descriptive analysis explored patients’ demographic and clinical characteristics within each altered management scenario. Continuous variables are presented by means (standard deviation, SD) or medians (interquartile range, IQR), categorical variables are presented by frequency (percentage). Calculations for each categorical variable were performed following the exclusion of missing values for this variable only. Non-parametric Mann–Whitney tests were performed to test for differences of medians between groups separately for each continuous and ordinal variable and Chi-squared tests for associations between nominal variables. To understand the national variation in the response to COVID-19, both in terms of MDT-M decisions and logistic arrangements, management decisions for the top 10 recruiting units (as exemplars) were compared. Analyses were computed using Stata MP (version 16). Where pertinent, the benefit of chemotherapy (without taking into account bisphosphonate treatment) using the *NHS Predict* online tool was calculated.^22^ Key exemplar healthcare costs (using unit costs from published databases [NHS Reference Costs and Supply Chain]) were compared between routine and ‘COVID-altered’ management to estimate potential financial impacts to the NHS (Supplementary Material).

### Data validity

We utilised REDCap’s built-in analysis tools to run tests of data completeness and consistency. In particular, the ‘phased’ data collection offered by the study design allowed us to perform validation checks to ensure consistency of data entry (Supplementary Table 3). Specifically, we calculated the percentage of concordance as the agreement of phases 1 and 2 of the study^18^ divided by total cases, for each category of ‘COVID-altered’ management.

## Results

**Fig. 2:**
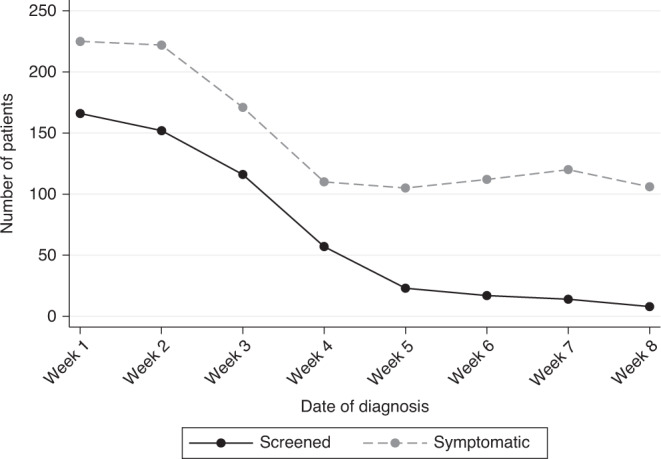
Change over time in the number of patients presenting with screen-detected versus symptomatic breast cancer during the study period. Week 1 begins on 16th March, and week 8 ends on 8th May.

There were 3776 patients included in the study from 64 breast units in the United Kingdom (with rapid data accrual and wide geographical representation from screening and symptomatic units and University Teaching Hospitals and District General Hospitals, Supplementary Fig. 1). Data validity tests showed high agreement in all fields interrogated, ranging from 95 to 99% (Supplementary Table 3). Of the patients included, 1530 (41%) had standard management and 2246 (59%) had ‘COVID-altered’ management at some point within their treatment journey. Patients with the higher-stage disease were statistically more likely to have ‘standard’ management (Table 1). Conversely, screen-detected cancers were more likely to have standard management, which may be a function of fewer screen-detected cancers being treated as the study period progressed (Fig. 2). Patients with ‘COVID-altered’ management had equivalent ER-positive disease (81%) and HER2-positive disease (12%) when compared to contemporary national data.^23^

**Table 1:** Patient demographics in the B-MaP-C study cohort.

	Standard management	‘COVID-altered’ management	*P* value	Total
	*N* = 1530	*N* = 2246		3776
Age (median-IQR)	56 (48–68)	60 (51–70)	<0.001	59 (50–69)
Missing	1	4		
T (*n* = 3682)				
Tis	119 (8%)	228 (10%)	<0.001	347
T1	577 (38%)	981 (45%)		1558
T2	609 (40%)	777 (36%)		1386
T3	159 (11%)	165 (8%)		324
T4	46 (3%)	21 (1%)		67
Missing	20	74		
N (*n* = 3666)				
N0/ N1mi	1037 (69%)	1665 (77%)	<0.001	2702
N1	318 (21%)	379 (18%)		697
N2	92 (6%)	74 (3%)		166
N3	63 (4%)	38 (2%)		101
Missing	20	90		
M (*n* = 3675)				
M0/MX	1493 (99%)	2149 (99%)	0.033	3642
M1	20 (1%)	13 (1%)		33
Missing	17	84		
WHO performance status (*n* = 3731)				
0	1215 (80%)	1688 (76%)	0.032	2903
1	187 (12%)	375 (17%)		562
2	76 (5%)	126 (6%)		202
3	36 (2%)	22 (1%)		58
4	6 (0%)	0 (0%)		6
Missing	10	35		
Presentation (*n* = 3716)				
Symptomatic	427 (28%)	913 (41%)	<0.001	1340
Screen-detected	1072 (72%)	1304 (59%)		2376
Missing	31	29		

*T* tumour stage, *N* nodal stage, *M* metastases, *WHO* World Health Organisation.

T, N are pathological TNM, except where patients were having ET/NACT, in which case TNM is taken from imaging, using T stage based on the largest reported size from all imaging modalities.

M1 = patients who were diagnosed with metastatic disease after surgery.

### Breast cancer management decisions altered due to COVID-19: neoadjuvant treatment

Of the 2216 patients who had MDT-M decisions in the preoperative setting, 252 patients had omitted (*n* = 160) or incomplete (*n* = 92) NACT. For those 160 patients where the pandemic resulted in the omission of NACT, the majority (143/156 [92%]) went on to receive ACT.

To allow postponement of surgery, 951 patients had ‘bridging’ ET (defined in the protocol as ‘Patient with hormone receptor-positive cancer having ‘bridging’ endocrine therapy due to a potential delay in surgery’), of which the vast majority (708/862, 82%) were postmenopausal (Table 2). Of patients commenced on ET, 740/774 (96%) were strongly ER-positive (Allred score 7–8), with only 4/774 (<1%) having a score below 5, and 140/900 (15%) assessed pre-operatively as node-positive. Although the primary reason for ‘bridging’ ET is likely due to anticipated reduced theatre capacity, in some, the decision may have been driven by comorbidity and increased risk of COVID-19 mortality.

**Table 2:** Patient and cancer-specific data for patients having selected major COVID-altered management decisions.

	Patients having omitted/incomplete NACT	Patients having ‘Bridging’ ET	Patients undergoing simple Mx when BCS possible	Patients not offered immediate breast reconstruction	Patients not having adjuvant CT	Patients not having adjuvant RT	Patients having five fractions RT
*N*	252^c^	951^c^	42^c^	299^c^	81^c^	96^c^	781^c^
Age^a^	52 (28–78)	65 (35–92)	59 (39–77)	50 (28–75)	63 (38–78)	69 (63–77)	59 (53–67)
Menopausal status (*n*)							
Pre/peri-menopausal	121 (50)	154 (18)	13 (33)	176 (61)	20 (25)	13 (15)	187 (26)
Postmenopausal	119 (50)	708 (82)	27 (67)	113 (39)	61 (75)	76 (85)	544 (74)
Missing	12	89	2	10	0	7	50
Tumour stage							
Is	0	87 (10)	0	23 (8)	0	16 (17)	46 (6)
1	58 (23)	455 (50)	15 (37)	80 (27)	33 (41)	57 (61)	433 (56)
2	142 (57)	310 (34)	23 (56)	132 (44)	39 (49)	15 (16)	252 (33)
3	40 (16)	50 (6)	2 (5)	58 (20)	6 (8)	4 (4)	40 (5)
4	10 (4)	4 (0)	1 (2)	4 (1)	2 (2)	2 (2)	3 (0)
Missing	2	45	1	2	1	2	7
Nodal stage							
0/mi	125 (50)	760 (84)	23 (56)	175 (59)	36 (45)	82 (86)	638 (83)
1	75 (30)	124 (14)	14 (34)	76 (26)	41 (51)	12 (13)	112 (15)
2	33 (13)	10 (1)	4 (10)	32 (11)	3 (4)	1 (1)	15 (2)
3	17 (7)	6 (1)	0 (0)	14 (5)	0 (0)	0 (0)	6 (1)
Missing	2	51	1	2	1	1	10
Metastases							
M0	248 (99)	897 (100)	41 (100)	293 (99)	80 (100)	95 (100)	770 (100)
M1	2 (1)	3 (0)	0 (0)	3 (1)	0 (0)	0 (0)	3 (0)
Missing	2	51	1	3	1	1	8
Size	MMG/USS	MMG/USS	MMG/USS	MMG/USS	Path.	Path.	Path.
Size in mm^b^	29·0 (1.2)	20·7 (0.5)	24·2 (2.2)	32·8 (1.5)	27·5 (1.8)	17·8 (1.4)	20·3 (0.6)
	28·3 (1.0)	16·9 (0.4)	23·7 (1.9)	23·9 (1.0)			
Missing	9/8	34/39	1/1	13/9	1	3	20
Histological type							
IDC	226 (92)	599 (65)	39 (95)	207 (71)	65 (82)	65 (71)	548 (72)
ILC	7 (3)	149 (16)	0 (0)	48 (16)	8 (10)	5 (5)	107 (14)
Mixed	4 (2)	31 (3)	1 (2)	9 (3)	3 (4)	1 (1)	26 (3)
Other	9 (4)	54 (6)	1 (2)	6 (2)	3 (4)	5 (5)	40 (5)
DCIS	0	94 (10)	0	232 (8)	0	16 (17)	45 (6)
Missing	6	24	1	6	2	1	15
Grade (invasive)							
1	2 (1)	204 (25)	1 (2)	14 (5)	3 (4)	19 (26)	126 (18)
2	67 (27)	507 (62)	14 (34)	147 (54)	42 (53)	43 (58)	407 (57)
3	178 (72)	109 (13)	26 (63)	110 (41)	35 (44)	12 (16)	181 (25)
Missing	5	37	1	5	1	3	67
Grade (DCIS)							
LG	0	12 (14)	0	1 (4)	0	0 (0)	0 (0.0)
IG	0	29 (33)	0	5 (23)	0	4 (27)	12 (27)
HG	0	46 (53)	0	16 (73)	0	11 (73)	33 (73)
Missing	0	7	0	1	0	1	0
ER							
+	121 (49)	922 (100)	24 (59)	221 (76)	59 (73)	76 (89)	656 (86)
−	127 (51)	3 (0)	17 (41)	71 (24)	22 (27)	9 (11)	104 (14)
Missing	4	26	1	7	0	8	21
PR							
+	78 (33)	620 (85)	19 (48)	160 (63)	48 (66)	51 (72)	462 (71)
−	155 (67)	106 (15)	21 (52)	95 (37)	25 (34)	20 (28)	188 (29)
Missing	19	225	2	44	8	21	221
HER2							
+	116 (47)	63 (8)	8 (20)	71 (26)	21 (26)	3 (4)	85 (12)
−	132 (53)	761 (92)	33 (80)	205 (74)	59 (74)	75 (96)	636 (88)
Missing	4	137	1	23	1	15	60
Ki67^b^	36·2 (3·6)	18·8 (2·5)	29·6 (5·1)	27·4 (3·4)	24·4 (6·0)	14.6 (2·5)	19.8 (1·6)
Missing	213	847	24	246	65	75	654
Comorbidities							
None	6 (2)	46 (5)	1 (2)	6 (2)	1 (1)	4 (4)	19 (2)
≥1	246 (98)	905 (95)	41 (98)	293 (98)	80 (99)	92 (96)	762 (98)
Missing	0	0	0	0	0	0	0
WHO PS							
0	197 (79)	667 (71)	28 (67)	261 (87)	63 (78)	61 (64)	658 (85)
1	40 (16)	180 (19)	9 (21)	29 (10)	14 (17)	26 (27)	104 (13)
2	11 (4)	74 (8)	5 (12)	9 (3)	4 (5)	7 (7)	13 (2)
3	2 (1)	13 (1)	0 (0)	0 (0)	0 (0)	2 (2)	2 (0)
4	0 (0)	0 (0)	0 (0)	0 (0)	0 (0)	0 (0)	0 (0)
Missing	2	17	0	0	0	0	4

*NACT*   neoadjuvant chemotherapy, *ET* neoadjuvant endocrine therapy, *Mx* mastectomy, *BCS*   breast-conserving surgery, *CT*   chemotherapy, *RT*   radiotherapy, *IDC*   invasive ductal carcinoma, *ILC*   invasive lobular carcinoma, *DCIS*   ductal carcinoma in situ, *LG*   low grade, *IG*   intermediate grade, *HG*   high grade, *ER*   oestrogen receptor, *PR*   progesterone receptor, *HER2*   Human epidermal growth factor receptor 2, *PS*   performance status.

^a^Median (IQR).

^b^Mean (SD).

^c^Patients could be in more than one category.

### Surgery

Preoperative imaging assessment was altered in 50 patients, for example with the omission of magnetic resonance imaging to assess disease extent. Of the 957 patients with altered surgical recommendations, 589/957 (62%) had breast-conserving surgery (BCS), 356/957 (37%) had a simple mastectomy and 12/957 (1%) had a mastectomy with IBR. There were 42 patients who had a simple mastectomy when BCS was clinically possible (defined in the protocol as MDT recommendation for simple mastectomy over BCS influenced by potential unavailability of radiotherapy), likely because breast radiotherapy could not be delivered locally. This group had a high number of premenopausal patients (13/40, 33%), with high-grade disease (26/41, 63% grade 3) and disproportionately more ER-negative disease (17/41, 41%) (Table 2), suggesting that oncological considerations impacted these decisions. Thirteen patients had change to standard practice because they had no completion axillary clearance (ANC) following a positive sentinel node, when ANC at that unit would have been offered pre-COVID. There is relative clinical uncertainty around completion ANC in subgroups of patients. For example, based on pre-COVID advice, all 13 patients were eligible for the POSNOC study^24^ where ANC could have been appropriately omitted. Nine patients also fulfilled Z0011 criteria for the omission of completion axillary clearance.^25^

The utilisation of IBR has doubled over the last two decades.^26^ However, in this cohort, there were 299 patients who were not offered IBR, when the usual pre-COVID practice would have included this. These patients were young (median age 50, 61% premenopausal), reflecting a subgroup where IBR would usually be considered a priority. The estimated cost of mastectomy and IBR for these patients would have been £1,636,969, whereas the total cost of mastectomy plus delayed reconstruction is an estimated £3,063,428 (an additional cost of £1,426,459; Supplementary Material).

### Adjuvant treatment

Of 1863 patients who had a post-operative MDT-M decision, 81 patients had adjuvant chemotherapy omitted, which would have been offered in the pre-COVID environment, including 13/81 patients with omitted NACT as described above. There were 62/81 patients in whom a decision was made to omit chemotherapy based on clinical grounds (without genomic testing). This group had a median benefit of chemotherapy of 3% (IQR 2–9%) using NHS Predict calculations.^22^ The majority of patients with omitted chemotherapy were postmenopausal (61/80, 75%), with no/low nodal burden (N0 or N1 = 77/80, 96%) and ER-positive disease (59/81, 73%) with one or more comorbidities (80/81, 99%), implying holistic risk-benefit decision- making (Table 2). Collaborating units were asked to report cases where genomic testing directly influenced MDT-M recommendation to avoid adjuvant chemotherapy (that, following NICE guidance, would not usually have been used pre-COVID). This was the case in 19/81 patients. In addition, extended-indication genomic testing outside of the 2018 NICE guidelines (such as patients with node-positive disease, which was outside of NICE guidance),^27^ was used in 27 patients, of whom 21 avoided chemotherapy based on genomic test outcome.

Thirteen patients with HER2-positive disease had no adjuvant-targeted (anti-HER2) therapy or chemotherapy when standard management would have included this. The median age of this group was 73 (range 52–84), with low tumour stage (T1 = 5/13, T2 = 7/13), low nodal stage (N0/N1 = 11/13) and largely ER-positive disease (11/13). Four patients with HER2-positive disease received adjuvant anti-HER2 therapy without systemic adjuvant chemotherapy, in accordance with COVID-19 guidance.

There were 96 patients in whom adjuvant radiotherapy was not recommended, whereas pre-COVID local MDT-M practice would have been to recommend radiotherapy. We explored the clinicopathological characteristics of these patients and noted that of these 96, 45 patients (47%) met inclusion criteria for the avoidance of radiotherapy based on NICE guidelines^6^ or the PRIME2 study.^28^ In line with the time guidelines for 5F breast and chest wall RT^12^ and the FAST-Forward publication, 781 patients had adjuvant radiotherapy with 5F where, pre-COVID, 15F would have been administered (Table 2). Of 64 units included in this study, 46 (72%) offered the fast-forward radiotherapy protocol, evidencing the ability to rapidly introduce new clinical practice based on high-quality trial data. As an exemplar of the potential positive health economic impact of changes to management, the cost saving of change to 5 F was investigated. The change from 15F to 5F for these patients is estimated to have saved £1,421,420 (supplementary material). Compared to those having 5F RT, 504 patients receiving 15F were younger (median age 56 years, IQR 49–67 vs 59 years, IQR 53–67, *P* = 0.006), had larger tumours (mean size 27.6 mm (1·1) vs 20.3 mm (0.6), *P* = 0.0000), and higher nodal burden (mean number of macrometastatic nodes 2.3 (0.2) vs 0.4 (0.1), *P* < 0.0001).

### Logistic changes to breast surgery during the COVID-19 pandemic

**Fig. 3:**
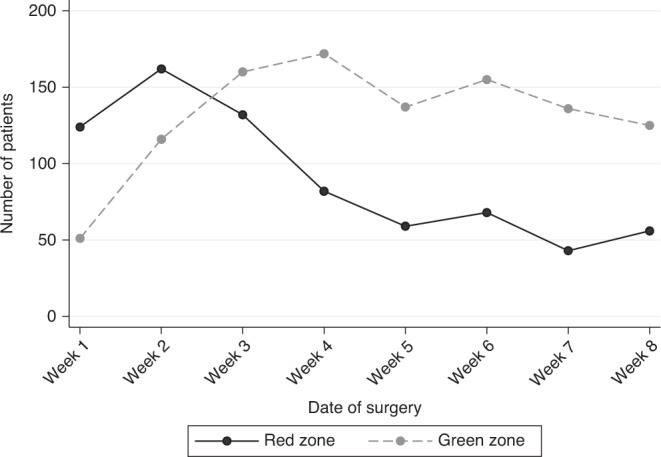
Transition to ‘green’ (COVID low-risk) theatres. Change over time during the ‘study period, showing an increase in the number of operations performed in a ‘green’ (COVID low-risk) versus ‘red’ (COVID high-risk) zone.

Of 3776 patients, 1778 (47%) had surgery within the study period; 1052 (59%) were in ‘green’ (COVID low-risk) zone operating theatre and 726 (41%) in ‘red’ (COVID high-risk) theatres. There was a move towards increasing access to ‘green’ theatres as the study progressed as units adapted their services in response to the pandemic, from 51 (29%) operations in the first week to 125 (69%) operations in the final week of the study (Fig. 3). The median time to surgery from diagnosis for patients (excluding those on NACT or ET) was 24 days (IQR 16–34), and was similar in both ‘red’ (25 days, IQR 15–33) and ‘green’ (24 days, IQR 16–34) theatres, which is within NHS Breast Screening Programme/NICE pre-COVID targets.

Recommended practice for sentinel lymph node biopsy surgery is with dual localisation using Technicium-99-m isotope and Patent blue dye.^29^ In 122 cases, sentinel node biopsy was performed with blue dye only. This was more common in ‘green’ theatres that were usually non-NHS independent sector hospitals (117/122, 96%) versus ‘red’ theatres that were usually NHS acute care trusts (5/122, 4%).

### Breast cancer management decisions: national variation

**Fig. 4:**
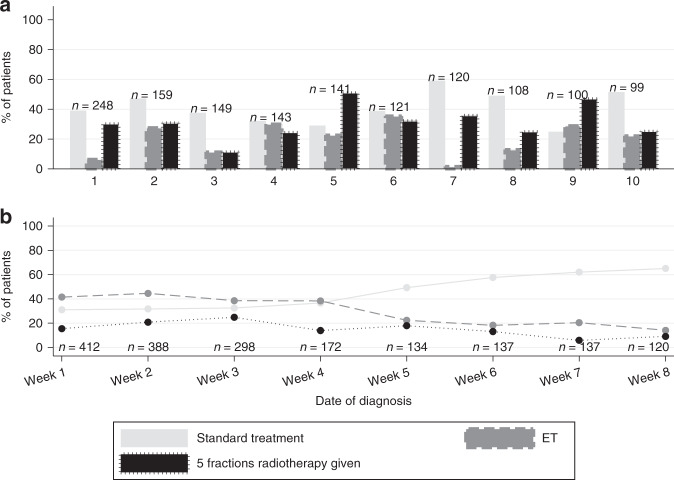
National Variations in management of breast cancer during the COVID-19 pandemic. **a** Variation in management decisions in the top 10 recruiting units, comparing the percentage of patients with (i) standard management (light grey bar), (ii) bridging ET (grey bar) and (iii) 5-fraction RT (black bar). **b** Trends in management decisions during the study period, by date of diagnosis at weekly intervals.

To get a sense of the variation in practice across the United Kingdom, we compared changes to normal practice in the ten units contributing the most patient datasets to the study (totalling 37% of the study cohort), with the frequency of ‘standard’ treatment, use of ‘bridging’ ET and use of five-fraction RT as exemplars. Within these ten units, the frequency of standard treatment ranged from 25% to 59%, the frequency of ET from 2% to 35% and of five fractions of RT from 11% to 51% of each hospital’s total patient cohort (Fig. 4a). This may highlight local differences in the ability to access theatre space or to rapidly implement a new evidence-based practice. There was an increase in the number of patients having ‘standard’ treatment and less use of ‘bridging’ ET as the study progressed (Fig. 4b), indicating some recovery in service delivery. In patients commenced on preoperative/’bridging’ ET, 210 patients went on to have surgery by the end of the study. The median length of time of ET for these patients was 22 days (IQR 15–31). The most common reason for proceeding with surgery early was unexpected availability of theatre capacity (53%).

### SARS-CoV-2 testing, outcome and impact on patient journey

There were 1392 patients tested for SARS-CoV-2, the majority in the preoperative setting (1033, 74%). Fourteen patients tested positive (1%). Of 11 patients testing positive before surgery, all were managed at home, without need for hospital admission, with a median time from diagnosis to surgery of 52 days (range 18–168). Those with >90-day delay (*n* = 2) were temporised with ‘bridging’ NET. There was an increase in preoperative hospital testing during the study period, no doubt, reflecting the increasing availability of SARS-CoV-2 tests nationally (Supplementary Fig. 2). Three patients tested positive post-operatively, of which two had surgery in a red zone.

There was no SARS-CoV-2-related mortality.

## Discussion

The COVID-19 pandemic has had unprecedented effects on healthcare provision across the United Kingdom. Early reports from both the United Kingdom and globally have shown that malignancy was a predictor of mortality^30,31^ and poor outcomes from COVID-19.^31–33^ No previous reports have focused specifically on alterations of care during the pandemic, to early breast cancer patients.^34^ The major challenges during the pandemic have been to weigh treatment benefit against potential transmission risk of SARS-CoV-2 to patients and healthcare providers, and to administer treatment in healthcare settings where resources and capacity are strained. The rapid publication of guidelines by several organisations^7–13,16^ facilitated the implementation of practical evidence-based decisions, as evidenced by the results of this study.

Despite treatment recorded as ‘COVID-altered’ in 2246 (59%) patients as per study definitions,^18^ the vast majority of patients in this study were treated according to pre-COVID guidelines. Only 439/2246 (19%) patients had a management that was clearly outside the pre-COVID breast cancer NICE guidelines (omission of a reconstruction or premenopausal patients on ‘bridging’ ET), implying that breast cancer oncological outcomes in this study are unlikely to be negatively impacted (although the psychological impact of reconstruction omission is yet to be determined). Where ‘COVID-altered’ management plans had to be instigated, these were mostly within guidelines published during the pandemic. There is likely, however, to be a cohort of women who are yet to present, either symptomatically or through screening, with breast cancer. Their outcomes may be disadvantaged through the late presentation, although that group lies outside the scope of this study.

Where theatre capacity has been an issue, there was increased use of preoperative ET compared to the usual pre-COVID practice,^29^ largely as ‘bridging’ therapy.^35^ Indeed, a large number of patients initially placed on ‘bridging’ ET in anticipation of significant surgical delay who have already had surgery, as theatre capacity, particularly in ‘green’ theatres, has increased. In those not receiving any neoadjuvant or bridging treatment (*n* = 1074), surgery has taken place in a timely manner. This has to be taken in context with the reduction in a number of patients presenting, particularly with screen-detected cancer. It is noteworthy that there were low rates of SARV-CoV-2 infection and no reported COVID-19-related post-operative deaths.

It is evident that some surgical decisions have reflected local resource availability for adjuvant therapies. For example, there was a cohort of patients (*n* = 42) undergoing simple mastectomy when pre-COVID practice would have been BCS followed by adjuvant RT. These patients were younger, with more aggressive tumour characteristics, and hence this represents, for a short period of time, a decision to prioritise reduction of recurrence risk, whilst there was uncertainty over RT availability. We have identified a large group of patients who have not had IBR when usual practice would have recommended this. UK NICE guidelines recommend all women undergoing a mastectomy be offered IBR.^6^ However, the COVID-19 pandemic has led to the suspension of IBR in many countries worldwide due to resource, workforce and safety concerns. This may lead to detrimental effects on the aspects of QoL such as body image.

Internationally, standard-of-care chemotherapy treatment regimens have been adapted to minimise the intensity of hospital visits and hospitalisation, and to prevent cancer treatment-induced complications of COVID-19.^7–11,13,15,16,20,36^ Transferring NACT to the adjuvant setting is oncologically safe,^37^ but may deprive the patient of the opportunity of downstaging to accommodate BCS, and prevent identification of nonresponders. These nonresponders may potentially be deprived of further adjuvant treatment such as the NICE-approved Trastuzumab emtansine in patients with HER2-positive disease or Capecitabine in patients with triple-negative breast cancer. The majority of patients omitting NACT received adjuvant chemotherapy reflecting the changing scenario during the COVID-19 ‘Alert Level 4’, where initial apprehension for systemic therapy had subsided by the time these patients had completed surgery.

In many units, there has been rapid adoption of the results of the FAST-Forward study, with an almost immediate change to treatment protocols across the country.^38^ We categorised 5F RT as ‘altered management’ because the start of the study predated the 5-year local control results of the FAST-Forward study, although prepublication national guidelines supported the use of 5F based on 3-year toxicity results of FAST Forward.^12^ This allowed us to determine the reactiveness of breast cancer services to new evidence and guidance issued during the pandemic. The 5F protocol was advantageous from a service provision perspective, where at the height of the pandemic, the workforce was greatly reduced, as well as from a safety viewpoint, given the reduced number of hospital visits and COVID-19 exposure risk. For those that did not have adjuvant radiotherapy, most were within criteria for planned avoidance of radiotherapy using NICE guidelines^6^ and PRIME2^28^ criteria. Had research trials been available, these patients may potentially have been recruited into the PRIMETIME study, which had been temporarily closed during the pandemic.

Inevitably, there are some limitations to a study of this nature, particularly one executed in such a short time frame. It is recognised that the data reported in this study have the potential to be subject to reporting bias, data entry error, and indeed, some decisions made early in the COVID-19 ‘alert level 4’ may have been subject to change particularly as the peak of the pandemic subsided. It is also appreciated that the study did not include all units in the United Kingdom, and not all participating units were able to collect data on consecutive patients within the study period, by the study deadline. Nevertheless, this still remains a representative national picture of the impact of the COVID-19 pandemic on breast cancer treatment in the United Kingdom.

Clinical research is a key component of high-quality breast cancer care. In the year 2018/19, over 9000 patients were recruited to breast cancer clinical trials (National Cancer Research Institute Annual Report 18/19). However, the pandemic has resulted in the suspension of many clinical trials, with the redeployment of cancer research staff to allow prioritisation of COVID-19 studies. Across all cancer sites, only 31% of cancer trials continued as planned during the pandemic (National Institute for Health Research (NIHR), personal communication, unpublished data). Although the NIHR has now published a framework for restarting clinical research, recovery of trial recruitment is slow. This has implications for patients in accessing trials and novel treatments, and for researchers to deliver studies to time and target. The extent of this impact on the UK breast cancer clinical research portfolio, however, remains to be elucidated.

Our study has described the extent of changes (and key cost implications) in the management of breast cancer in the United Kingdom during the COVID-19 pandemic on a patient and population-based level, as a response to timely and feasible guidance that was largely followed at a time of national crisis. Appraisal of these changes will guide the evaluation of the impact of the pandemic on immediate patient outcomes and the degree to which breast cancer management has been affected in routine clinical practice. This will assist with planning of service delivery once routine breast cancer management resumes and in the event of a further pandemic. There are significant implications for the COVID-19 ‘recovery’ phase with patients on endocrine therapy requiring cancer resection, and those denied IBR awaiting delayed breast reconstruction. Anticipating the recommencement of breast screening, this delayed (or postponed) activity is likely to overstretch breast surgical services. In the long term, we will have the opportunity to assess the impact of treatment alterations on the rate of disease recurrence and overall patient survival, QoL and the impact of these management decisions on the service and health economics.

## Supplementary information

Supplementary material BJC B-MaP-C

## Data Availability

Data supporting the results reported in the paper cannot be found on publicly available databases. The data have been uploaded by collaborators onto a RedCap database, and individual units have access to their own data, but not the overall national data.
