# Publisher Correction: Breast cancer management pathways during the COVID-19 pandemic: outcomes from the UK ‘Alert Level 4’ phase of the B-MaP-C study

**DOI:** 10.1038/s41416-021-01465-z

**Published:** 2021-06-23

**Authors:** Rajiv V. Dave, Baek Kim, Alona Courtney, Rachel O’Connell, Tim Rattay, Vicky P. Taxiarchi, Jamie J. Kirkham, Elizabeth M. Camacho, Patricia Fairbrother, Nisha Sharma, Christopher W. J. Cartlidge, Kieran Horgan, Stuart A. McIntosh, Daniel R. Leff, Raghavan Vidya, Shelley Potter, Chris Holcombe, Ellen Copson, Charlotte E. Coles, Ramsey I. Cutress, Ashu Gandhi, Cliona C. Kirwan, on behalf of the B-MaP-C study collaborative

**Affiliations:** 1 The Nightingale Breast Cancer Centre, Wythenshawe Hospital, Manchester University NHS Foundation Trust, Manchester, M23 9LT UK; 2 Department of Breast Surgery, St. James’s University Hospital, Leeds, LS9 7TF UK; 3 Department of Surgery and Cancer, Imperial College, London, UK; 4 Department of Breast Surgery, The Royal Marsden NHS Foundation Trust, Downs Road, Sutton, Surrey SM2 5PT UK; 5 Leicester Cancer Research Centre, Clinical Sciences Building, University of Leicester, Leicester, LE2 2LX UK; 6 Division of Population Health, Health Services Research, and Primary Care, School of Health Sciences, University of Manchester, Manchester, M13 9PL UK; 7 Trustee, Independent Cancer Patients Voice, Manchester, UK; 8 Breast unit, Level 1 Chancellor wing, St James’s Hospital, Leeds, LS9 7TF UK; 9 Queen Margaret Hospital, Dunfermline, Whitefield Rd, Dunfermline, KY12 0SU UK; 10 Patrick G Johnston Centre for Cancer Research, Queen’s University Belfast, 97 Lisburn Road, Belfast, BT9 7AE UK; 11 The Royal Wolverhampton NHS Trust, Wolverhampton Road, Wolverhampton, WV10 0QP UK; 12 Bristol Centre for Surgical Research, Population Health Sciences, Bristol Medical School, Canynge Hall, Whatley Road, Bristol, BS8 2PS UK; 13 Bristol Breast Care Centre, North Bristol NHS Trust, Southmead Road, Bristol, BS10 5NB UK; 14 Linda McCartney Centre, Royal Liverpool and Broadgreen University Hospital, Prescot Street, Liverpool, L7 8XP UK; 15 Cancer Sciences Academic Unit, University of Southampton and University Hospital Southampton, Tremona Road, Southampton, SO16 6YD UK; 16 Department of Oncology, University of Cambridge, Cambridge, UK; 17 Division of Cancer Sciences, School of Medical Sciences, Faculty of Biology, Medicine and Health, University of Manchester, Oglesby Cancer Research Building, Manchester Cancer Research Centre, Wilmslow Road, Manchester, M20 4BX UK

**Keywords:** Breast cancer, Surgical oncology, Health care economics, Quality of life, Health policy

Correction to: *British Journal of Cancer* 10.1038/s41416-020-01234-4, published online 25 March 2021

The original version of this article unfortunately contained a mistake. The author metadata of this article had to be corrected due to an error in typesetting. The publisher apologises for the mistake. The original article has been corrected.

