# Supplementary material for: Breast cancer management pathways during the COVID-19 pandemic: outcomes from the UK ‘Alert Level 4’ phase of the B-MaP-C study
Source: Br J Cancer. 2021 Mar 25;124(11):1785–94. doi: 10.1038/s41416-020-01234-4 (PMC7993073; doi:10.1038/s41416-020-01234-4)
Supplement: Supplementary file 1 — Supplementary material BJC B-MaP-C [file 41416_2020_1234_MOESM1_ESM.docx]

**Supplementary material 1**

Table S1. Recommendations from the Association of Breast Surgery

| If operating theatre capacity was limited, patients should be prioritized for surgery in the following order: oestrogen receptor (ER) negative cancers, human epidermal growth factor receptor 2-positive (HER2+) cancers, pre-menopausal ER+ cancers, post-menopausal ER+ cancers, high grade ductal carcinoma in situ (DCIS), intermediate or low grade DCIS. In case of insufficient theatre capacity, post-menopausal ER+ patients could be commenced on endocrine therapy. |
| --- |
| Neoadjuvant chemotherapy should only to be offered to patients with inoperable disease and should not be used to downstage from mastectomy to breast conserving surgery (BCS) or to perform axillary conservation in patients with ER- or HER2+ disease. This is to avoid a potential delay to definitive surgical treatment of the primary cancer. |
| All immediate breast reconstruction (IBR) to be suspended, with delayed reconstruction to be offered at a later date once the service returned to normal to minimise surgical complexity, length of stay, complication risks, therefore reducing the risk of developing COVID-19. |
| Genomic testing on the core biopsy should be considered in all grade 3 or node positive ER+ patients.^6^ Patients with a high recurrence score should be advised to have surgery as they would ordinarily need adjuvant chemotherapy. |

Table S2. Summary of breast cancer treatment recommendations from other relevant organisations

| **Organisation** | **Summary of recommendations** |
| --- | --- |
| NHS England ^8^ | - Categorisation of patients into priority levels for surgery, systemic anti-cancer therapy, and radiotherapy (RT). - Separation of the location of emergency from elective operations within the same trust. - Consider non-surgical treatment options if the outcomes are similar. - Consider alternative and less resource-intensive treatment regimens. |
| National Institute for Health and Care Excellence (NICE) ^9,^ ^36^ | - Prioritisation of systemic anticancer treatments (adjuvant or neoadjuvant) into priority level 1 to 6 depending upon the chance of success. - Aim to alter delivery of systemic anti-cancer treatments in order to reduce immunosuppressive side effects, and to minimise hospital visits. |
| Royal College of Radiologists (RCR)^12^ | - Omit RT for patients >65 years old with invasive cancer <30mm, grade 1 or 2, ER+HER2- and node negative, who are planned for endocrine therapy (ET). - Deliver RT in 5 fractions only for patients with node negative cancers. - Boost RT should be omitted unless 40 years old or younger. - Nodal RT can be omitted in post-menopausal women for T1 ER+HER2- grade 1 or 2 tumours with 1 or 2 macrometastasis. |
| Cancer Core Europe (CCE) consortium ^7^ | - Multidisciplinary team meeting to consider alternative treatment modalities with the fewest visits or lowest capacity problems or that are shortest in duration. - Consider hypofractionated RT for patients with limited additional benefits of regular regimens. - For surgery, consider postponing surgeries with a potential for high morbidity and mortality. |
| European Society of Medical Oncology ^10^ | Patient management categorized into high, medium, and low priority;   - High: Patients with invasive breast cancer where biology and stage will drive management. Chemotherapy (neoadjuvant or adjuvant) recommended for patients with triple negative and HER2 positive cancers. - Medium: Patients with non-invasive breast cancer. In addition, patients with stage I/II ER+PR+HER2- low grade can be considered for ET with delayed surgery. Consider the use of hypofractionated RT. - Low: Patients with increased risk of developing breast cancer. |
| COVID 19 Pandemic Breast Cancer Consortium ^17^ | Categorisation of breast cancer patients into priority levels (A,B,C) for urgency of care;   - Priority A: Patients with conditions that are immediately life threatening or symptomatic requiring urgent treatment. - Priority B: Patients with conditions that do not require immediate treatment but should start before the pandemic is over. The decision for neoadjuvant chemotherapy was left with each institution. For T2 or N1 ER+HER2- cancers, ‘bridging’ ET can be considered. Hypofractionated RT regimens should be considered. - Priority C: Patients with conditions that can be safely deferred until the pandemic is over. This includes patients diagnosed with DCIS and T1N0 ER+HER2- cancers. |
| European Society of Breast Imaging ^11^ | - Women who have symptoms suspicious of breast cancer should undergo regular diagnostic work up - Women with an indication for needle biopsy should undergo this procedure as soon as possible - Women with breast cancer requiring evaluation of ongoing neoadjuvant therapy should undergo these studies without further delay - Asymptomatic women who have been informed of the suspension of breast screening should schedule their check within 3 to 6 months of the due date. |
| Coronavirus (COVID-19): clinical guidelines for cancer treatment, Scotland ^40^  28^th^ April 2020 | Categorisation of patients according to priority   - consider non-surgical options, including prolongation of neoadjuvant treatment and non-surgical treatment if the outcomes are similar   General approach to prioritising patients on systemic anti-cancer therapy:   - Categorise patients by treatment intent and risk-benefit ratio associated with treatment. - Consider alternative and less resource-intensive treatment regimes. - Seek alternative methods to monitor and review patients receiving systemic therapies. - Consider whether systemic therapies can be given in alternative regimens, different locations or via other modes of administration to minimise patient exposure and maximise resources |

Table S3. Data validation studies. The data entered during phase 1 of data entry was compared to data entered during phase 2 of the study ^19^. Concordance percentage was calculated for each category where indicated.

| **Phase 1** | **Phase 2; pre-COVID standard management** | **Phase 2; ‘COVID-altered’ management** | **% Concordance** |
| --- | --- | --- | --- |
| Omitted neoadjuvant chemotherapy, when standard management would have included this | Neoadjuvant chemotherapy | - Surgery first - Neoadjuvant endocrine therapy - Primary endocrine therapy | 98.9%  (2168/2192) |
| Patient having incomplete or altered neoadjuvant chemotherapy | Neoadjuvant chemotherapy | Neoadjuvant chemotherapy, but incomplete or altered | 99.4%  (2180/2192) |
| Patient with hormone receptor positive cancer having bridging endocrine therapy due to a potential delay in surgery | Surgery first | - Neoadjuvant endocrine therapy - Primary endocrine therapy | 96.3%  (2111/2192) |
| Simple mastectomy in a patient whose standard therapy would otherwise have been breast conservation followed by adjuvant radiotherapy | Breast conserving surgery | Simple mastectomy | 99.1%  (1993/2011) |
| Simple mastectomy performed, with a view to delayed reconstruction, in a patient who would have been offered immediate reconstruction | Skin sparing or nipple-sparing mastectomy and immediate reconstruction | Simple mastectomy | 96.3%  (1936/2011) |
| Patient who would usually have adjuvant radiotherapy, but have not been offered this | Adjuvant Radiotherapy | No Adjuvant Radiotherapy | 98.4%  (1787/1816) |
| Patient who would usually have adjuvant radiotherapy, but have not been offered this | Adjuvant Radiotherapy | Yes, but hypofractionation (usually, 5 fractions) | 94.9%  (1724/1816) |
| Patient who would usually have adjuvant chemotherapy, but have not been offered this | Adjuvant Chemotherapy | - No Adjuvant Chemotherapy - No, based on Oncotype DX score | 97.6%  (1877/1924) |


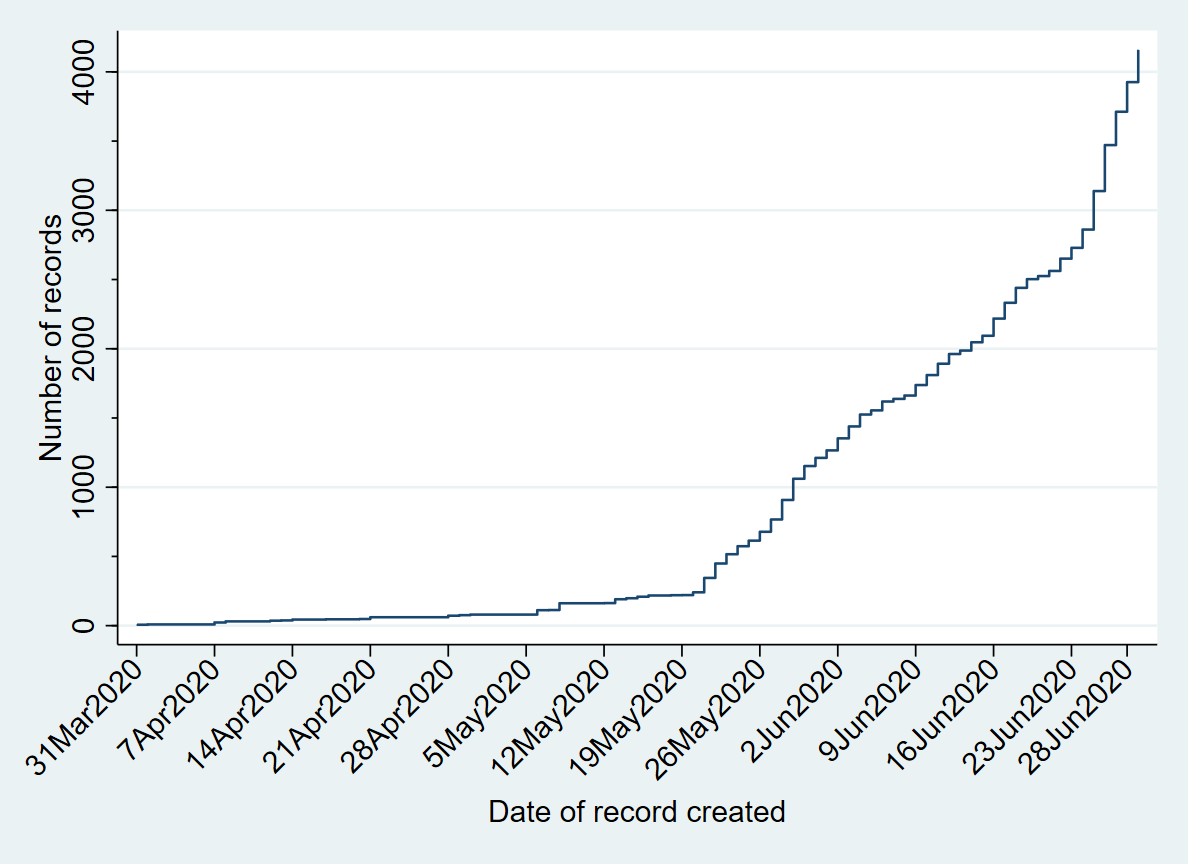


Figure s1a. Graph of data accrual (date record created on REDCap) over the 13-week period of the study, from its launch on 31^st^ March 2020.


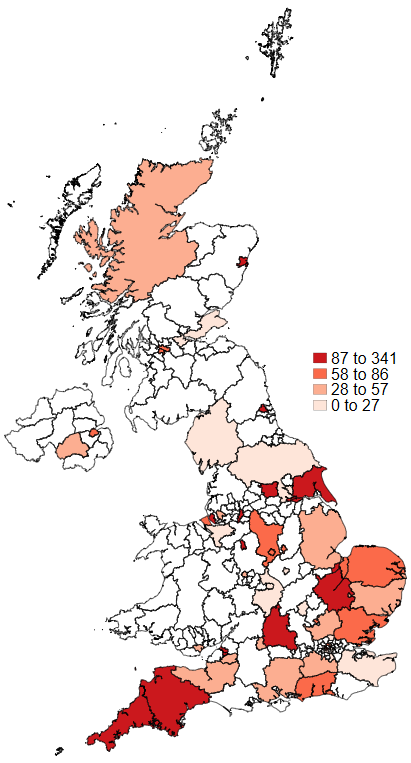


Figure s1b. Spatial map of patient recruitment per county based on hospital postcode; legend breaks defined by quartiles of patients recruited (excluding Isle of Man and ROI)


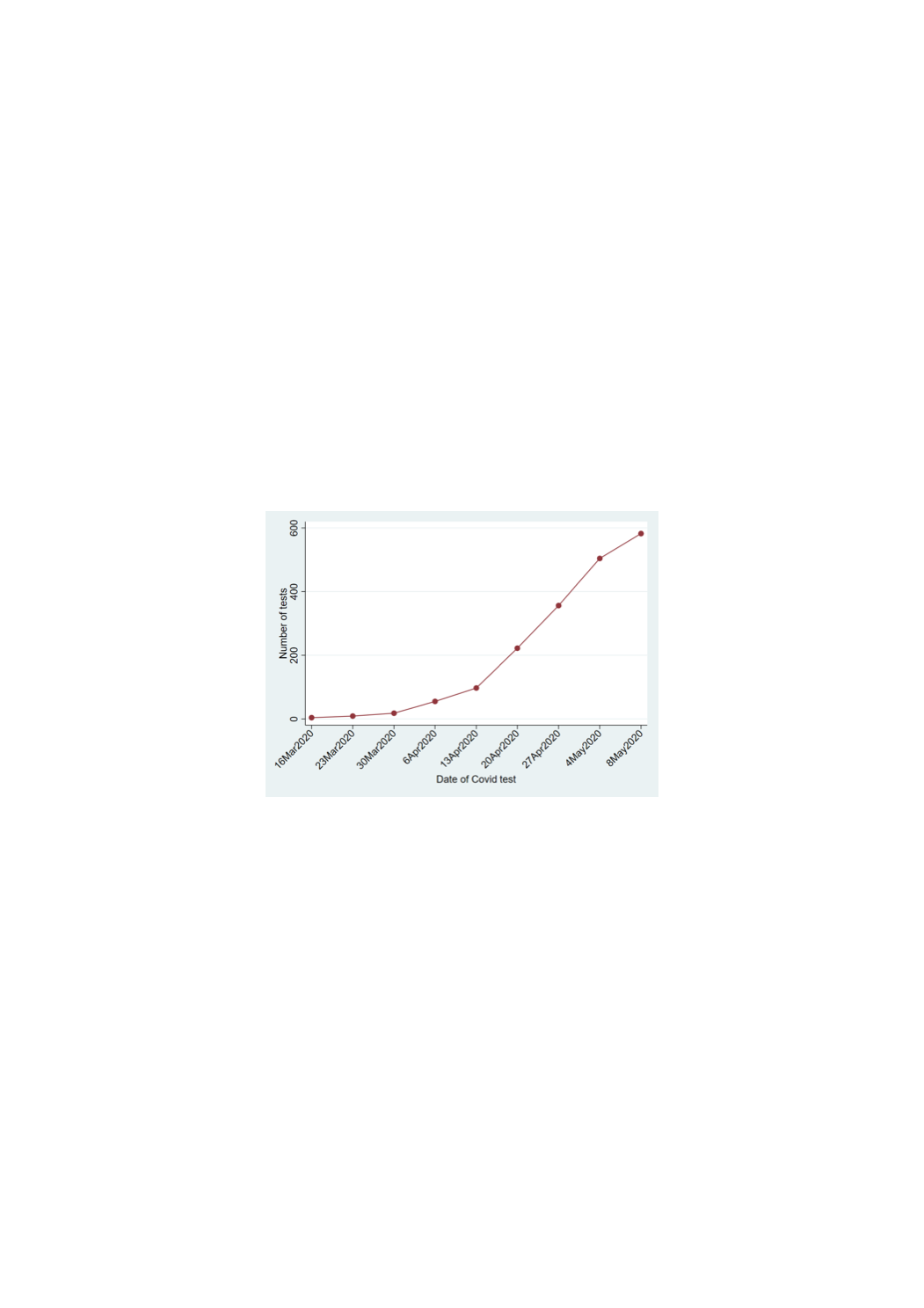


Figure s2. Temporal change in the uptake of SARS-CoV-2 testing during the study period.

**Supplementary: Health Economics**

**Short-term economic implications of impact of COVID-19 on breast cancer management**

**Background**

The key potential cost implications of the impact of COVID-19 on breast cancer management, from the perspective of the UK NHS, were identified as hypofractionated radiotherapy (RT) and delayed breast reconstruction. The costs implications of these changes were estimated for the patients included in the B-MaP-C study.

**Methods**

The resource use associated with standard and hypofractionated RT and breast reconstruction were identified through consultation with clinical experts. The unit costs associated with these resources were identified using published databases (NHS Reference Costs (1) and Supply Chain (2)) (see Table S3). Costs were estimated for patients whose care was altered due to COVID-19 (i.e. those receiving hypofractionated RT and delayed reconstruction) during the ‘alert phase’ of the B-MaP-C study. Costs were estimated for the management they would have received pre-COVID-19 (i.e. standard RT and immediate reconstruction) and for the management they either did receive (hypofractionated RT) and are likely to receive (delayed reconstruction). To estimate pre- and post-COVID costs of breast reconstruction, data from published UK-based observational studies was used to estimate the proportion of patients who undergo different reconstructive procedures in the immediate and delayed settings. These data were used to estimate the proportion of patients in the study who would have undergone the different procedures had they had an immediate reconstruction. These data were also used to estimate the proportion of patients who would undergo the different procedures now that they were going to be conducted in the delayed setting (see Table S4).

**Table S4.** Unit costs and estimated resource use of standard and altered management

|  | Unit cost | Source |
| --- | --- | --- |
| *Standard RT**  1 preparation session  15 fractions delivered | £1095  £2,730 | NHS reference costs 2018/19 (1) |
| *Hypofractionated RT**  1 preparation session  5 fractions delivered | £1095  £910 | NHS reference costs 2018/19 (1) |
| *Immediate reconstruction*  Single-stage implant reconstruction  Single-stage implant reconstruction (with ADM)  2-stage implant reconstruction  2-stage implant reconstruction (with ADM)  Pedicled reconstruction (with/without implant)  Perforator flap reconstruction | £2,542  £4,798  £5,084  £7,340  £5,909  £12,396 | NHS reference costs 2018/19 (1)  NHS Supply chain (July 2020) (2) |
| *Delayed reconstruction*  Mastectomy  2-stage implant reconstruction  2-stage implant reconstruction (with ADM)  Pedicled reconstruction (with/without implant)  Perforator flap reconstruction | £2,542  £5,084  £7,340  £5,706  £11,103 | NHS reference costs 2018/19 (1)  NHS Supply chain (July 2020) (2) |
|  | % undergoing procedure | Source |
| *Immediate reconstruction*  Implant  Pedicled  Perforator flap  Single-stage implant  2-stage implant  Single-stage implant (without ADM)  Single-stage implant (with ADM)  2-stage implant (without ADM)  2-stage implant (with ADM) | 54%  25%  21%  78%  22%  26%  74%  54%  45% | Mennie et al (3)  Potter et al (4)  Potter et al (4)  Potter et al (4) |
| *Delayed reconstruction*  2-stage implant  Pedicled  Perforator flap  2-stage implant reconstruction (without ADM)  2-stage implant reconstruction (with ADM) | 24%  34%  42%  77%  23% | Mennie et al (3)  Mennie et al (3) |

*£182 per fraction; ADM = acellular dermal matrix

**Results**

There were 781 patients who received hypofractionated RT. Had they received 15 fractions this would have cost £2,987,325, whereas the cost to deliver the 5-fraction course was £1,565,905, which is a saving of £1,421,420.

The estimated costs of breast reconstruction in the immediate and delayed scenarios are reported in Table S5. For the 299 patients who were not offered immediate reconstruction the estimated total cost of their mastectomies and delayed reconstructive surgeries is £1,426,459 more than the estimated cost of had the reconstructions been done immediately.

**Table S5.** Estimated costs of immediate and delayed breast reconstructions

|  | Immediate | Delayed | Difference in costs |
| --- | --- | --- | --- |
| Cost of reconstructions | £1,636,969 | £2,303,370 | £666,401 |
| Cost of mastectomies | £0* | £760,058 | £760,058 |
| TOTAL cost | £1,636,969 | £3,063,428 | £1,426,459 |

*cost of mastectomy included in cost of immediate reconstruction

Limitations of cost analysis

It was necessary to make a number of assumptions in order to estimate the costs associated with breast reconstruction. For example, it was assumed that all patients who did not have an immediate reconstruction would still opt for reconstruction after a delay. A single cost was used for all reconstructions which used ADM (based on the cost of Strattice™ which is a biological mesh) however the brand and type of mesh (e.g. biological or synthetic) is likely to be different in different hospitals. Data to quantify this was not identified and so it is unknown to what extent this cost has been over or underestimated. Furthermore, data were not identified on the proportion of delayed two-stage implant-based reconstructions in which ADM is typically used. Two source studies were identified which reported the proportion of immediate two-stage implant-based reconstructions in which ADM was used (23% (3) or 46% (4)). Expert clinical opinion was sought from breast surgeons in the UK who agreed that the use of ADM in the delayed setting was likely to be low, therefore the smaller of the two estimates from the source studies was used (3). Current practice in breast reconstruction changes rapidly over time, however the source studies used to inform the cost estimates used data from over 5 years age and so may not be a true reflection of current practice.

There are two key economic considerations which have not been included in the estimates presented here. Firstly, direct (e.g. fuel, travel) and indirect (e.g. lost earnings, time off work) costs saved from the patient’s and their informal caregiver’s perspective of only having to travel to receive 5 fractions of RT rather than 15. Secondly, any medium- or long-term economic impacts on recovery and QoL of having to wait for breast reconstruction.

REFERENCES

1. NHS Improvement. National schedule of reference costs.

2. NHS Supply Chain. NHS Supply Chain - Catalogue [Internet]. 2020. Available from: https://my.supplychain.nhs.uk/catalogue

3. Mennie JC, Mohanna PN, O’Donoghue JM, Rainsbury R, Cromwell DA. National trends in immediate and delayed post-mastectomy reconstruction procedures in England: A seven-year population-based cohort study. Eur J Surg Oncol. 2017 Jan 1;43(1):52–61.

4. Potter S, Conroy EJ, Cutress RI, Williamson PR, Whisker L, Thrush S, et al. Short-term safety outcomes of mastectomy and immediate implant-based breast reconstruction with and without mesh (iBRA): a multicentre, prospective cohort study. Lancet Oncol. 2019 Feb 1;20(2):254–66.

**Supplementary: Data analysis plan**

**Study collaborators, data accrual, and validity**

Patients recruited into the study

This could include figures and consort diagram which will help focus the reader of the different denominators in the study.

Figure suggestion: study schematic

Figure suggestion: Recruitment timeline.

Figure suggestion: Date of diagnosis vs Surgery by presentation (symptomatic vs screen-detected)

Data Validity

Comment on using RedCap tools to check completeness and validity of data entry

Contact units when discrepancy – i.e. M1 patients, diagnosis date before mid-2019

Concordance between section 2 and 3 – potentially S concordance / ICC test to look at section 2 vs section 3 – contact units where discrepancy lies.

**MDT management decisions and demographics of patient cohort**

Select only patients diagnosed between 01/08/2019 – 08/05/2020 (ensuring patients entering at the correct time – so those having NACT and having adjuvant decisions are captured).

For consistency, and clarity- timelines in the paper (displayed in some way on consort-style figure) are:

- date of diagnosis as above for all patients in the study

- date of diagnosis in the ‘alert phase’ decisions – 16/03 – 08/05

- date of surgery in the alert phase (for e.g. trends of green vs red) – 16/03 – 08/05

- How many had altered management?

Summarise demographics as per table 1 below.

The geographical variation in management decisions – consider displaying data for 10 largest units in graphical form.

Figure suggestion. Geographical variation - Dots/heat map of city and numbers recruited

|  |  | Standard Management | COVID-altered management | Total | p (X^2^, Mann Whitney)) |
| --- | --- | --- | --- | --- | --- |
| Age  (median and range) |  |  |  |  |  |
| T (n=1827) | Tis |  |  |  |  |
|  | T1 |  |  |  |  |
|  | T2 |  |  |  |  |
|  | T3 |  |  |  |  |
|  | T4 |  |  |  |  |
| N (n=1610) | N0 / Nmi |  |  |  |  |
|  | N1 |  |  |  |  |
|  | N2 |  |  |  |  |
|  | N3 |  |  |  |  |
| M (n=1825) | M0 / MX |  |  |  |  |
|  | M1 |  |  |  |  |
| WHO performance status (n=1861) | 0 |  |  |  |  |
|  | 1 |  |  |  |  |
|  | 2 |  |  |  |  |
|  | 3 |  |  |  |  |
|  | 4 |  |  |  |  |
| Presentation | Symptomatic |  |  |  |  |
|  | Screen-detected |  |  |  |  |
|  |  |  |  |  |  |
| T, N are pathological TNM, except where patients are having NET/NACT  M1= patients who were diagnosed with metastatic disease after surgery. | | | | | |

Table 1- demographics of patient cohort.

**‘COVID-Altered’ management scenarios**

Descriptive patient- and cancer- specific data on sub-cohorts, as per fields in table 2.

- Omitted NACT, ‘Incomplete’ or altered NACT (group these together)

- Bridging ET

Operative:

- Simple Mx when BCS possible

- Simple MX when immediate recon possible

Post-operative:

- Patient not adjuvant radiotherapy

- Patient having radiotherapy with 5 fractions

- Patient not having adjuvant chemotherapy,

- Patients not having adjuvant targeted (e.g. herceptin) therapy

What not to report in table (small print), so leave out / mention in text only

- Patient who would usually have margin re-excision surgery for close margins (based on local protocols), who do not have further surgery.

- Patient who would usually have completion axillary clearance for sentinel node macro-metastases, who do not have further surgery

- Patient who would usually have a sentinel node biopsy for incidental invasion found during surgery for DCIS, but has not had this.

Suggested table 2: patient- and cancer - specific data based on altered management category

|  | OMITTED/  Incomplete NACT | Patients having ET | Patients having Simple MX when BCS was possible | Simple Mx instead of Immediate recon | Patients not having adjuvant Chemo | Patients not having adjuvant RT | Patients having 5 fractions RT |
| --- | --- | --- | --- | --- | --- | --- | --- |
| N= |  |  |  |  |  |  |  |
| Age (median and range) |  |  |  |  |  |  |  |
| Menopausal status: |  |  |  |  |  |  |  |
| Pre-menopausal |  |  |  |  |  |  |  |
| Peri-menopausal |  |  |  |  |  |  |  |
| Post-menopausal |  |  |  |  |  |  |  |
| T |  |  |  |  |  |  |  |
| is |  |  |  |  |  |  |  |
| 1 |  |  |  |  |  |  |  |
| 2 |  |  |  |  |  |  |  |
| 3 |  |  |  |  |  |  |  |
| 4 |  |  |  |  |  |  |  |
| N |  |  |  |  |  |  |  |
| 0/Mi |  |  |  |  |  |  |  |
| 1 |  |  |  |  |  |  |  |
| 2 |  |  |  |  |  |  |  |
| 3 |  |  |  |  |  |  |  |
| M1 |  |  |  |  |  |  |  |
| size (in mm, mean and 95% CI) |  |  |  |  |  |  |  |
| Histological type |  |  |  |  |  |  |  |
| IDC |  |  |  |  |  |  |  |
| ILC |  |  |  |  |  |  |  |
| Mixed |  |  |  |  |  |  |  |
| Other |  |  |  |  |  |  |  |
| grade |  |  |  |  |  |  |  |
| 1 |  |  |  |  |  |  |  |
| 2 |  |  |  |  |  |  |  |
| 3 |  |  |  |  |  |  |  |
| DCIS |  |  |  |  |  |  |  |
| LG |  |  |  |  |  |  |  |
| IG |  |  |  |  |  |  |  |
| HG |  |  |  |  |  |  |  |
| ER |  |  |  |  |  |  |  |
| Pos |  |  |  |  |  |  |  |
| neg |  |  |  |  |  |  |  |
| PR |  |  |  |  |  |  |  |
| pos |  |  |  |  |  |  |  |
| neg |  |  |  |  |  |  |  |
| her2 |  |  |  |  |  |  |  |
| pos |  |  |  |  |  |  |  |
| neg |  |  |  |  |  |  |  |
| ki67  Mean (95% CI) |  |  |  |  |  |  |  |
| Nodal macromets  Median (range) |  |  |  |  |  |  |  |
| Comorbidities |  |  |  |  |  |  |  |
| None |  |  |  |  |  |  |  |
| >1 |  |  |  |  |  |  |  |
| WHO Performance score |  |  |  |  |  |  |  |
| 0 |  |  |  |  |  |  |  |
| 1 |  |  |  |  |  |  |  |
| 2 |  |  |  |  |  |  |  |
| 3 |  |  |  |  |  |  |  |
| 4 |  |  |  |  |  |  |  |

Report above categories in figure vs Standard over time across the study (x-axis = 16/03 to 08/05) to assess for a pattern or a recovery, and if there was a lag whilst centres made alternative arrangements.

Figure suggestion: Trends in management decisions during the study period, by date of diagnosis between 16/03/2020 and 08/05/2020.

**COVID-altered management - Pre-operative setting**

A) Neo-adjuvant chemotherapy.

- Number of patients not having NACT THAT WOULD USUALLY HAVE IT

- For those that didn’t get NACT – how many got adjuvant chemotherapy?

- If Her2+ve, did they get anti-Her2 (at any stage in treatment)? Define group

- How many having anti-HER2 without chemo?

B) ET

- Describe (shown in table 2) specifically the group of patients - size / nodal status, age / menopausal status and ER status +/- Allredscore (% that had 0-4 vs 5-6 vs 7-8)

- What treatment – Tamoxifen / Letrozole etc.

- For the pre-menstrual patients on ET – were these smaller, node negative cancers?

- Was there a geographical variation in the top 10 units – usage of ET ranged from X% to X% - to show that access to theatre varied.

- How many went on to have surgery? Within what time frame?

**COVID-altered management - Surgery**

A) Where/when was surgery performed?

- How many in in ‘green’ and in ‘red’ theatres - display as with trends over time frame - 16/3 – 08/05 in figure.

- The median (IQR) time from date of diagnosis to date of surgery in those patients that had surgery first (excluding those patients that had ET/NACT)

- The time to surgery in green vs red (suggested figure)

- Report % done in green / red in top 10 recruiting hospitals – to show national variation

B) What surgery was performed?

- Details for surgery performed e.g. X% had BCS, Y% had simple mastectomy, and Z% had a skin/nipple sparing mastectomy with immediate reconstruction. Note: we only have this data for the ‘altered’ group, not the standard group.

- Patients that had simple mastectomy when standard therapy would otherwise have been breast conservation followed by adjuvant radiotherapy – define group demographics using radiological (pre-op) size and other patient- and cancer-specific data as per fields in table 2.

- Compare to ‘standard’ data = NABCOP and NHSBSP data

- Group of patients that had a simple mastectomy when immediate reconstruction would have been usually offered – define, using radiological (pre-op) size and other patient- and cancer-specific data as per fields in table 2

- Compare to NMBRA and IBRA2 data

- Sentinel node biopsy was not performed using dual-technique - use of a Patent-V blue dye only (with no radioisotope) was seen in X% of circumstances when operated in a ‘green’ zone vs X% in a ‘red’ zone.

- Standard = surgery QPI S2a- screening only and NICE

- Compare group that didn’t have completion clearance for low-risk disease to the Z011/POSNOC criteria.

**COVID-altered management - Adjuvant**

A) Patients not having adj Chemo

- Describe based on path TNM, receptors, px age, px comorbidity (as per table 2) and % gain based on NHSpredict

- If no chemo – was this due to Oncotype use?

- Oncotype used outside of NICE 2018 guidelines – look at number used, and briefly define group (all node positive?) – did they all get chemotherapy?

Note: those not getting adjuvant Chemo – some would be Her2+ve, and may have got anti-Her2 only

Reference: NICE guidelines for chemotherapy, and UKBCG priorities list; NICE rapid guidelines COVID and Cancer- interim use of SACT

B) Patients not having adjuvant radiotherapy

- No RT group: define as per table 2: age, path size, grade, receptors

- Compare to PRIME2/NICE – ie what % were within guidelines for omission of RT.

C) Patients having 5-fractions RT

Use of 5F RT: rapid adaptation of 5 fractions (FF) protocol adaptation

- Figure suggestion: use of 5F RT over study period

- Out of X units in the study, X% were offering 5-fractions RT.

- Look at patients who still had 15F RT and compare to 5F group, using data as per table 2.

Reference: NICE/FF/RCR guidelines.

**SARS-CoV2 testing and outcome**

- How many tested? And How many positive pre-op and post-op?

- Uptake of pre-operative testing

- Figure suggestion- pre-operative testing increased with time (date of surgery – 16^th^ March – 5^th^ May)

- What was the impact of a COVID diagnosis? Delay in surgery? HDU?

**Health Economic Impact of the management decisions.**

Potential questions:

a) Fast tracked 5F – less money spent on RT

b) Less money spent and resources utilised for immediate reconstruction (cost savings were – use known data for frequency of implant vs DIEP)

c) Eventual Cost of delayed recon – high DIEP %
